# The Use and Abuse of Transcranial Magnetic Stimulation to Modulate Corticospinal Excitability in Humans

**DOI:** 10.1371/journal.pone.0144151

**Published:** 2015-12-02

**Authors:** Martin E. Héroux, Janet L. Taylor, Simon C. Gandevia

**Affiliations:** 1 Neuroscience Research Australia, Randwick, NSW, Australia; 2 University of New South Wales, Randwick, NSW, Australia; University of Ottawa, CANADA

## Abstract

The magnitude and direction of reported physiological effects induced using transcranial magnetic stimulation (TMS) to modulate human motor cortical excitability have proven difficult to replicate routinely. We conducted an online survey on the prevalence and possible causes of these reproducibility issues. A total of 153 researchers were identified via their publications and invited to complete an anonymous internet-based survey that asked about their experience trying to reproduce published findings for various TMS protocols. The prevalence of questionable research practices known to contribute to low reproducibility was also determined. We received 47 completed surveys from researchers with an average of 16.4 published papers (95% CI 10.8–22.0) that used TMS to modulate motor cortical excitability. Respondents also had a mean of 4.0 (2.5–5.7) relevant completed studies that would never be published. Across a range of TMS protocols, 45–60% of respondents found similar results to those in the original publications; the other respondents were able to reproduce the original effects only sometimes or not at all. Only 20% of respondents used formal power calculations to determine study sample sizes. Others relied on previously published studies (25%), personal experience (24%) or flexible post-hoc criteria (41%). Approximately 44% of respondents knew researchers who engaged in questionable research practices (range 32–70%), yet only 18% admitted to engaging in them (range 6–38%). These practices included screening subjects to find those that respond in a desired way to a TMS protocol, selectively reporting results and rejecting data based on a gut feeling. In a sample of 56 published papers that were inspected, not a single questionable research practice was reported. Our survey revealed that approximately 50% of researchers are unable to reproduce published TMS effects. Researchers need to start increasing study sample size and eliminating—or at least reporting—questionable research practices in order to make the outcomes of TMS research reproducible.

## Introduction

Transcranial magnetic stimulation (TMS) is a popular technique in neuroscience. Its popularity stems from the allure of drawing important inferences about human brain function with a seemingly non-invasive, and certainly non-painful technique with few serious side effects [1, 2]. Originally introduced as a technique in clinical neurophysiology to assess central and peripheral motor conduction [3] and subsequently motor cortical physiology (e.g., [4]), TMS has blossomed into a method to explore brain physiology. It comes in many styles: from single pulses to complex sets of repetitive pulses, several of which have gained favour as methods to assess and modulate particular aspects of cortical function [5, 6]. These include paired associative stimulation [7] and various forms of repetitive stimulation (such as theta burst stimulation, e.g. [8]).

In our experience some of the published protocols work but they are often difficult to replicate routinely (e.g. [9]). Our colleagues frequently tell us at conferences that they also have difficulty reproducing the original published effects. These difficulties may in part reflect the small effects being investigated—usually in a small number of subjects—which results in low statistical power [10]. These issues are compounded by the presence of physiological and non-physiological factors that can further limit the reproducibility of published findings [10–17]. The apparent success of these techniques may be fuelled by the bias to publish positive rather than negative results [18–20], which can lead to questionable research practices [21–25] and inflated effect sizes [10, 26–30].

To determine whether our experience reflects that of the broader research community, we conducted on online survey to determine the prevalence of non-reproducible results in published and non-published studies that have used TMS to alter motor cortical or corticospinal excitability in humans. To gain insight into the cause of this low reproducibility, survey respondents were also questioned on various research practices known to contribute to low statistical power and exaggerated effect sizes.

## Methods

To gain an overview of the field using TMS to probe or alter motor cortical and corticospinal excitability in humans, we invited the first and last authors of relevant publications to complete an anonymous internet-based survey (see S1 File) approved by the University of New South Wales Health Sciences Ethics Board. An initial search was conducted in PubMed in February 2014 for studies that had used TMS or one of its common variants to modulate motor cortical and corticospinal excitability (see S2 File). References (n = 1,486) were reviewed by one of the authors (MH) and those that were clearly not relevant were excluded. The email addresses of first and last authors of the remaining references were obtained from the manuscript or by web search. A total of 153 researchers were invited to take part in the survey. After completing the survey, researchers were entered into a draw—independently conducted by the local IT department—to win an iPad.

In brief, the survey asked about the number of years respondents worked in the field and the type of TMS protocols they had previously used. We then asked about their published and unpublished studies that involved TMS and, in particular, how study sample sizes were determined and whether the results were in line with the original published findings. Finally, we asked respondents how they thought other researchers perform and report TMS studies and, using the same questions, we asked how they themselves performed and reported TMS studies. On completing the survey, respondents were invited to provide additional comments.

A sub-sample of papers were reviewed to determine whether the questionable research practices listed in our survey [Q8] are routinely reported in the literature. To obtain a random and representative sample, papers focusing on theta-burst stimulation published between 2010-Oct 2014 were identified and digital copies obtained (see S2 File). A total of 56 papers were retained. Each paper was reviewed by one of the authors (SG) to determine whether the questionable research practices listed in our survey were reported and results were verified by a second author (MH).

## Results

Of 153 invited researchers who use TMS to modulate corticospinal excitability we received 47 completed surveys (see S3 File). Respondents, who could select more than one research area, worked in a variety of fields: neuroscience (19.1%), motor control (21.0%), neurophysiology (21.7%), clinical neurology (15.9%), rehabilitation (14.0%) and psychology (4.5%) [Q1]. They had been working in these fields for a mean of 14.2 years (95% confidence interval 12.1–16.4; range 1–30 years) [Q2] and had published a career mean number of 46.6 papers (CI 30.1–65.3) using TMS to study the human motor system [Q4]. A subset of these papers (16.4, CI 10.8–22.0) used TMS techniques specifically to alter corticospinal excitability. When asked about relevant file-drawer papers (i.e. studies that were completed but not published), all but 9 of the respondents had at least one such paper, with the mean being 4.0 papers (CI 2.5–5.7) [Q4]. Three respondents reported large numbers of file-drawer papers (10, 15 and 30 papers), which exceeded their number of published TMS-related papers.

Respondents had experience with a variety of TMS techniques, and several respondents had experience with more than one technique. Of the many forms of TMS, repetitive TMS at low frequency (<1Hz) and high frequency (>1Hz) were commonly used (19.7% and 19.1%), as was paired associative stimulation (20.1%). Slightly less frequent were intermittent (16.3%) and continuous (16.3%) theta burst stimulation [Q3].

A range of methods were used by respondents to determine sample size in TMS studies [Q5]. Respondents could select more than one method. Of the 140 responses, only 20% indicated that formal power calculations were used; a greater number indicated a reliance on previously published studies (25%) or personal experience (24%). A further 15% indicated that sample size was set prior to the start of the study, but additional subjects were tested if needed. In 5% of cases the sample size was adjusted based on how the data were looking. The remaining responses indicated that sample size was set prior to start of the study, but fewer subjects were tested because a clear effect was (5.7%) or was not (5.7%) observed.

For the various TMS protocols they had used, we attempted to gauge whether investigators were able to reproduce a similar effect to what was reported in the original studies [Q6]. The percentage of respondents who answered yes was 61% for paired associative stimulation, 45% for continuous theta burst stimulation, 45% for intermittent theta burst stimulation, 60% for low frequency (<1 Hz) and 59% for high frequency (>1 Hz) repetitive TMS. The size of the observed effects was either smaller (32 responses), larger (3 responses) or the same as those by the original studies (64 responses). The remaining respondents were able to reproduce these effects only sometimes (56 responses) or not at all (18 responses). This applied to the majority of respondents who had used either form of theta burst stimulation. Respondents who were unable to reproduce an effect similar to that which was originally published were twice as likely to stop using the TMS protocol than seek to publish the negative results (12 *vs* 6 reports). Also, there was no difference in respondents’ years of experience and whether they were able or unable to reproduce published results (Wilcoxon rank sum test, p = 0.441).

Finally, we asked whether respondents used practices that could increase the chance of finding statistically significant results. Response rates for these questionable practices are presented in Table 1. On average, 44% of respondents knew researchers who engaged in these practices (range 30–68%), whereas only 18% admitted to these practices (range 6–38%). Almost 70% of respondents knew researchers who screened their subjects to identify those that responded in a predictable way to various TMS protocols. Fewer respondents admitted to this practice. Among the other questionable practices, 13–21% of respondents had previously failed to report all the experimental conditions from a study, had selectively reported data from sub-sample, of subjects or had rejected data based on a ‘gut’ feeling or without statistical justification. There was nearly total agreement (45 of 47 respondents, 96%) that these sorts of practices should be reported in publications.

**Table 1:** Prevalence of questionable research practices.

**Questionable research practices**	**Others [count(%)]**	**Self [count(%)]**
Screen for ‘responders’ to a TMS protocol	38 (68)	18 (38)
Drop data points based on a gut feeling	18 (38)	6 (13)
Exclude data after looking at impact on results	14 (30)	3 (6)
Not report all experimental conditions	19 (40)	10 (21)
Selectively report outcomes	23 (49)	5 (11)
Selectively report time points	14 (30)	5 (11)
Selectively report sub-groups of subjects	18 (38)	8 (17)
Reject ‘outliers’ without statistical support	19 (30)	10 (21)

See S1 File for the exact wording used in the online survey.

Across the 56 papers that were reviewed, none of the questionable research practices noted above were explicitly reported. While the majority of studies monitored background EMG, only four provided clear criteria on the time-period and threshold level used to exclude trials. Only one study included a sample size calculation and several studies did not provide the gender or handedness of subjects.

Several researchers volunteered comments about the survey and their experience using TMS, some of which are highlighted here. One researcher commented on the potential effect of the survey results:

“Thank you for carrying out this study of research practices with TMS. In my opinion it is an area in which real results have become difficult to distinguish from noise due to sloppy research practices (driven by a pressure to publish) and positive publication bias in most journals. It has reached a point where it is very difficult to design any neuromodulation study because the positive control conditions taken from previous results are simply not replicable, almost without exception.”

Another researcher commented on the difficulty to publish negative results and the long-term impact this has on one’s career choice:

“After all my experiences with TMS and troubles publishing negative/smaller results during my PhD, I decided to shift my research career to another subject. I hope something will change in reporting and interpretation of the TMS (but also direct current stimulation) results and the techniques can be of use for some patient groups.”

As a final example, a researcher described their experience with repetitive TMS to improve motor function in patients:

“I perform repetitive TMS for the purpose of functional restoration. In my experience, the traditional 10-session protocol is too weak to induce a robust result. […] I answer this survey based on the observation on traditional 10-session repetitive TMS.”

## Discussion

An anonymous internet-based survey was sent to researchers who had been first or last authors on published papers in which TMS was used to alter motor cortical excitability. The response rate was 31% and many of the respondents had been using TMS for more than a decade. They provided information on research results obtained using a variety of popular TMS techniques and detailed how these studies were designed and carried out. Results from our survey highlight the difficulty experienced by researchers to reproduce published research results. In addition, we found evidence that researchers in this field engage in, but fail to report, questionable research practices.

When questioned about TMS-induced effects on motor cortical excitability, 45–60% of respondents indicated they had success, to a greater or lesser extent, reproducing the original published results. Others were able to reproduce the original results only sometimes (32%) or not at all (10%). While this could be seen as evidence TMS protocols work in certain circumstances, this interpretation may well be wrong. It is well established the neuroscience research is dogged by small sample size, low statistical power and true effects that are often small [10]. Hence the risk of false discoveries is high [18] which causes the size of reported effects to be exaggerated [26, 27] and likely an over-optimistic picture of reproducibility. While some studies and expert reviews have acknowledged the high variability in responses to non-invasive brain stimulation and attempted to isolate controllable factors which determine the variability such as prior motor activity, attention, time of testing, age, and gender [11–13, 16, 17], the effect of publication bias, low statistical power and questionable research practices has been largely, if not totally, ignored. Based on our respondents’ reports, up to half of all studies, and hence published papers, in this research field should fail to reproduce the original results. However, such statements are rare in the literature and this biased representation contributes to the false view that TMS-induced effects are robust.

Our survey reveals a varied and somewhat haphazard approach to determining study sample size. About half of respondents relied on personal experience or previously published studies. In about one in five cases, formal sample size calculations were performed, possibly with exaggerated effect sizes from the literature. In a similar fraction of cases, additional subjects were tested based on ad-hoc analyses, or fewer subjects were tested because a clear effect was or was not present. Thus, a sub-set of researchers keep an eye on evolving probability values, a practice that violates a key tenet of a priori statistical testing [31]. Regardless of the approach used, sample sizes are often too small in neuroscience research and this results in low statistical power [10]. All studies can be affected, including those trying to reproduce previously published effects. For example, by testing the same number of subjects as a study that reported barely significant results, you have as much chance of rejecting the same null hypothesis as you do correctly calling a coin flip [10].

But how do studies with small sample sizes discover statistically significant effects? When statistical power is low, the first study to publish an effect is often the most biased towards an extreme result—the winner’s curse [32]. Subsequent studies are often less biased and find evidence of smaller or even contradictory effects [27]. Extreme effects tend to occur when thresholds such as statistical significance are used and they are most severe when studies are too small and thus have low statistical power [10]. For example, the original report on theta-burst stimulation involved only 9 subjects and concluded: ‘Here we describe a very rapid method of conditioning the human motor cortex using rTMS that produces a controllable, consistent, long-lasting, and powerful effect on motor cortex physiology and behavior after an application period of only 20–190 s’ [8]. In stark contrast to these *consistent* and *powerful* effects, the same research group conducted a subsequent study involving 52 subjects and concluded: ‘The cTBS and iTBS after-effects were highly variable. Indeed in this set of participants there was no overall effect of either form of stimulation’ [33]. In support of these latter findings, a recent study involving 56 subjects found no overall effect for PAS, iTBS or direct current stimulation [11]. Approximately 40% of subjects responded as expected to each of the stimulation techniques and only 12% of subjects responded in this way to all three techniques. The take-home message is clear: researchers should be wary of new published effects, especially when sample size is small. To establish reproducibility for an effect, a large sample size is mandatory.

There are other, more insidious factors that can bias research results towards extreme values. *Questionable research practices* is an established term that describes an unhealthy flexibility in collecting and analysing research data. These practices have been documented in many fields (e.g. medicine [23]; psychology [34]) and their incidence is believed to be increasing [21]. Why are such practices so problematic? Computer simulations of experimental data reveal that questionable research practices markedly increase false discovery rates by increasing the likelihood of finding a statistically significant effect (e.g. [24]). In line with previous reports [23, 34], our survey found questionable research practices were not uncommon among researchers. Between 6 and 38% of respondents reported using such practices, whereas 2–3 times as many believed that other researchers in the TMS field used questionable research practices. This mismatch between respondents’ and others’ practices could represent a bias in reporting, or could also be explained by a bias in the sample of respondents who may be especially concerned about questionable research practices, as they elected to complete the survey. The response rate to our survey was similar to that of other web-based surveys [35, 36]. However, it remains to be determined whether our results capture the opinions and experiences of the entire TMS field. The most common questionable practices reported in our survey were to exclude data, select subjects who are known to respond to a TMS technique, choose outcomes and time points, and select data from a sub-group of the main cohort. There was a uniform belief among respondents that such practices should be reported in published reports. In contrast to this sentiment, examination of 56 papers from our original sample found no mention of any questionable research practices. It is unfortunate that these dubious practices which predispose to false positive results appear to be so pervasive that they may constitute everyday practice.

Publication bias—where significant results are more likely to get published—has a long history in science [37]. Approximately half the respondents to our survey indicated they could not reproduce the original TMS effects: is it any wonder we also found evidence of a large file-drawer problem? Many TMS studies are not written up or accepted for publication and end up in a real or metaphorical file-drawer, never to be seen again [20, 27, 38]. This behaviour is well documented across branches of science and has been well analysed in sociology [19, 39], psychology [40, 41] and medicine [42]. While we were surprised by the numbers of dormant papers—an average of 4 per respondent—we were staggered that ∼ 15% of respondents had more papers in their file-drawer than papers published in the TMS literature. This reporting bias needs to be addressed if we are to determine the true effect of TMS techniques. Also worrying are the written comments we received indicating that this reporting bias also impacts the design of clinical interventions and the careers of individual researchers who feel pressured to publish positive results.

**Fig 1:**
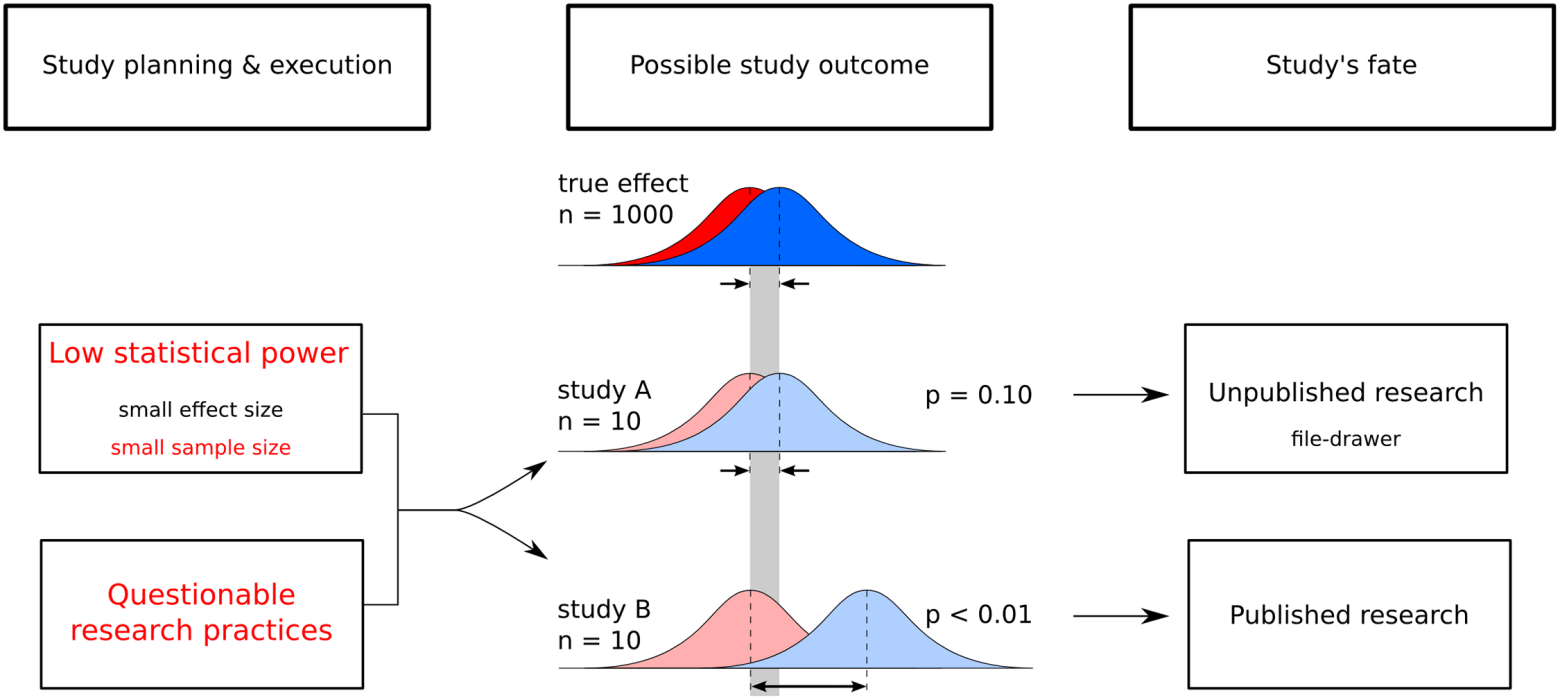
Factors contributing to irreproducible TMS results. When planning and executing a research study, the size of the investigated effect and the size of the sample directly influence a study’s statistical power—probability of correctly rejecting the null hypothesis when the null hypothesis is false—and the certainty of reported researcher results. Selecting sample size based on previous experience, published reports or power calculations based on inflated effect sizes from the literature often results in too few subjects being tested. In a study with low statistical power, significant results (*i.e. p* < 0.05) are biased towards extreme values(i.e. a large effect; study B). Independently, questionable research practices will also increase the rate of false discoveries and exaggerated effect sizes. Because these results meet the traditional level of statistical significance, they will likely become part of published literature. For the unlucky scientist who did not find statistically significant results (study A), the study may never be written up or it will be rejected by publishers because it presents uncertain, negative results. These studies become part of the cemetery of unpublished scientific research, the file-drawer.

A diagram was developed to summarise our findings on the irreproducibility of published TMS results (Fig 1). While not intended to represent all research conducted in this field, the diagram can help identify possible changes to improve the current state of TMS research (indicated in red font). First, researchers have to stop questionable researcher practices. The most severe practices, such as rejecting data based on a gut feeling, have no place in science. The less severe practices are more widespread, with some possibly becoming common practice. Regardless of their perceived severity, questionable research practices tend to not be reported. However, without these details reproducible TMS research will never become a reality [43]. Second, researchers have to stop conducting small studies with questionable sampling practices. The evidence can no longer be ignored and the benefits of larger sample sizes should be welcomed. We should increase the certainty of reported effects [10, 32] rather than doing so cosmetically by using standard error bars on graphs [44]. By increasing the level of certainty surrounding the size of an investigated effect, readers and editors will be interested regardless of the *positiveness* or *negativeness* of results, thus doing away with the arbitrary p-value and the file-drawer [32].

Irreproducible research, publication bias, and questionable research practices have become increasingly worrisome to researchers, policy makers, and journal editors [21, 30, 43, 45, 46]. Our survey reveals that research using TMS to alter motor cortical excitability is not immune to these problems. Commonly, survey respondents were only sometimes able or never able to reproduce the original reported effects, and they and their colleagues engaged in various questionable research practices. While a cure for these large-scale, endemic problems has yet to be found, we believe that increasing study samples size and eliminating—or at least reporting—questionable research practices would be a simple step towards more reproducible TMS research. This is important because TMS research is increasingly being translated into clinical practice, and patients and their physicians have the right to expect that potential therapies are based on sound and reproducible research.

## Supporting Information

S1 File Survey. Complete survey questions as they appeared to survey respondents.
(PDF)

S2 File Medline search strategy. (PDF)

S3 File Survey results. Spreadsheet containing all results from the survey.
(XLS)
