# Correction: The Use and Abuse of Transcranial Magnetic Stimulation to Modulate Corticospinal Excitability in Humans

**DOI:** 10.1371/journal.pone.0147890

**Published:** 2016-01-21

**Authors:** Martin E. Héroux, Janet L. Taylor, Simon C. Gandevia

There is an error in the 10^th^ sentence of the Abstract. The correct sentence is: Approximately 44% of respondents knew researchers who engaged in questionable research practices (range 30–81%), yet only 18% admitted to engaging in them (range 6–38%).

There is an error in the third and fourth sentences of the fifth paragraph in the Results. The correct sentences are: On average, 44% of respondents knew researchers who engaged in these practices (range 30–81%), whereas only 18% admitted to these practices (range 6–38%). About 80% of respondents knew researchers who screened their subjects to identify those that responded in a predictable way to various TMS protocols.

There are errors in Table 1. Please see the correct Table 1 here.

**Table 1:** Prevalence of questionable research practices.

Questionable research practices	Others [count(%)]	Self [count(%)]
Screen for ‘responders’ to a TMS protocol	38 (81)	18 (38)
Drop data points based on a gut feeling	18 (38)	6 (13)
Exclude data after looking at impact on results	14 (30)	3 (6)
Not report all experimental conditions	19 (40)	10 (21)
Selectively report outcomes	23 (49)	5 (11)
Selectively report time points	14 (30)	5 (11)
Selectively report sub-groups of subjects	18 (38)	8 (17)
Reject ‘outliers’ without statistical support	19 (40)	10 (21)

See S1 for the exact wording used in the online survey.
