# Supplementary material for: The Use and Abuse of Transcranial Magnetic Stimulation to Modulate Corticospinal Excitability in Humans
Source: PLoS One. 2015 Dec 2;10(12):e0144151. doi: 10.1371/journal.pone.0144151 (PMC4668054; doi:10.1371/journal.pone.0144151)
Supplement: S1 File — Complete survey questions as they appeared to survey respondents. (PDF) [file pone.0144151.s001.pdf]

* This survey is anonymous and no information will be linked back to you or your e-mail address.

THE USE OF TMS TO MODULATE CORTICOSPINAL EXCITABILITY

1. What area of research do you work in? Please select all that apply.
   □ Psychology
   □ Neuroscience
   □ Motor control
   □ Clinical neurology
   □ Rehabilitation
   □ Neurophysiology
   □ Other, please specify

2. Approximately how long have you used transcranial magnetic stimulation (TMS) to evaluate the corticospinal system in humans (e.g., MEP amplitude, MEP latency, silent period, input-output curves)?

   Number of years: □ (please enter a numerical value between 0 and 30)

3. Have you used any of the following TMS protocols to alter corticospinal excitability induce neuroplastic changes in human subjects? Please select all that apply.
   □ Paired associative stimulation (PAS)
   □ continuous theta-burst stimulation (cTBS)
   □ intermittent theta-burst stimulation (iTBS)
   □ low-frequency repetitive stimulation (rTMS; ≤ 1Hz)
   □ high-frequency repetitive stimulation (rTMS; > 1Hz)

Please identify additional protocols you have used that are not listed (please separate each protocol name using a comma):

* This survey is anonymous and no information will be linked back to you or your e-mail address.

4. **A)** Approximately how many studies have you published in peer-reviewed journals that have used TMS to investigate the human motor system?

   Number of publications: ☐ (please enter a numerical value between 0 and 999)

   B) How many of these publications have specifically used a TMS protocol to alter corticospinal excitability?

   Number of publications: ☐ (please enter a numerical value between 0 and 999)

   C) Approximately how many studies have you done but not published that have used a TMS protocol to alter corticospinal excitability?

   Number of publications: ☐ (please enter a numerical value between 0 and 999)

5. When you have used a TMS protocol to modulate corticospinal excitability in a study, how was the sample size of the study determined? Please select all sampling strategies that you have used.

   □ Using previously published results to perform a power calculation and estimate sample size requirements.
   □ Based on prior personal experience.
   □ Based on sample size of published studies.
   □ Based on how the data are looking.
   □ Prior to starting the study, but stopping early if no clear effect is noted.
   □ Prior to starting the study, but allowing for additional subjects to be included if needed.
   □ Prior to starting the study, but stopping early if a clear effect is noted.

* This survey is anonymous and no information will be linked back to you or your e-mail address.

6. *(This question will have sub-questions that depend on the measure(s) selected in question 3. It will ask the main question (6A) for each of the stimulation protocols identified in question 3. For each sub-question, a follow-up question will be asked depending on whether 'yes', 'no' or 'sometimes' was selected. If 'yes' was selected, question 6B will be asked. If 'no' is selected, question 6C will be asked. If 'sometimes' was selected, a text box will be provided and the respondent invited to explain.)*

A) When using [*insert chosen fields from question 3, including each 'additional' protocol listed in text box*], have you been able to reproduce a similar effect (e.g., facilitation, inhibition) to what was reported in the original literature?

□ Yes
□ No
□ Sometimes

B) If you were ***able*** to reproduce an effect, was it similar in magnitude to the original published effect?

□ Yes
□ No, the effect found in our laboratory was smaller
□ No, the effect found in our laboratory was larger

Additional comments:

C) If you were ***not able*** to reproduce an effect, what steps did you take? Please select all that apply.

□ Collect data from a greater number of subjects.
□ Select a subset of subjects that were 'susceptible' to the investigated effect.
□ Contact the original authors for clarification of the published protocol.
□ Stop using the stimulation protocol.
□ Modify the stimulation protocol.
□ Publish the finding that you were not able to reproduce the published effect.

Additional comments:

* This survey is anonymous and no information will be linked back to you or your e-mail address.

7. Please answer **Yes** or **No** to the following questions:

	Yes	No
Are you aware of other researchers that screen subjects based on whether they are considered 'responders' to a certain TMS protocol?	□	□
Are you aware of other researchers that drop observations or data points from analyses based on a gut feeling that they were inaccurate?	□	□
Are you aware of other researchers that decide whether to exclude data after looking at the impact of doing so on the results?	□	□
Are you aware of other researchers that do not report all of a study's experimental conditions in research publications?	□	□
Are you aware of other researchers that selectively report outcomes in research publications?	□	□
Are you aware of other researchers that selectively report time points in research publications?	□	□
Are you aware of other researchers that selectively report sub-groups of subjects in research publications?	□	□
Are you aware of other researchers who visually inspect their data and reject trials or subjects deemed to be 'outliers' without the support of statistical analysis?	□	□
Should these various practices be reported by researchers when they publish their research results?	□	□

* This survey is anonymous and no information will be linked back to you or your e-mail address.

8. Please answer **Yes** or **No** to the following questions:

	Yes	No
Have you ever screened subjects based on whether they are considered 'responders' to a certain TMS protocol?	◻	◻
Have you ever dropped observations or data points from analyses based on a gut feeling that they were inaccurate?	◻	◻
Have you ever decided whether to exclude data after looking at the impact of doing so on the results?	◻	◻
Have you ever not reported all of a study's experimental conditions in research publications?	◻	◻
Have you ever selectively reported outcomes in research publications?	◻	◻
Have you ever selectively reported time points in research publications?	◻	◻
Have you ever selectively reported sub-groups of subjects in research publications?	◻	◻
Have you ever visually inspected data and rejected trials or subjects deemed to be 'outliers' without the support of statistical analysis?	◻	◻

Thank you for your participation in this survey. If you have any additional comments related to the use of TMS to modulate corticospinal excitability in human subjects, please do so in the box provided below.

Additional comments:
