# Supplementary material for: The Use and Abuse of Transcranial Magnetic Stimulation to Modulate Corticospinal Excitability in Humans
Source: PLoS One. 2015 Dec 2;10(12):e0144151. doi: 10.1371/journal.pone.0144151 (PMC4668054; doi:10.1371/journal.pone.0144151)
Supplement: S2 File — (PDF) [file pone.0144151.s002.pdf]

Medline search criteria to identify potential researchers to invite to take part in the survey

(TMS OR rTMS OR transcranial magnetic stimulation OR PAS OR paired-associative stimulation OR theta burst stimulation OR TBS[Title/Abstract])

AND

(motor OR corticospinal OR corticomotor[Title/Abstract])

AND

(modulation OR neuromodulation OR plasticity OR neuroplasticity[Title/Abstract])

AND

(human)

Medline search criteria to identify potential published studies on theta-burst stimulation that would be reviewed for the prevalence of reporting questionable research practices.

(theta burst stimulation OR TBS OR iTBS[Title/Abstract])

AND

(intermittent[Title/Abstract])

AND

(motor OR cortical[Title/Abstract])

AND

(human)
